# Effects of habitat factors on the plant diversity on naturally-restored wind farm slopes

**DOI:** 10.7717/peerj.14912

**Published:** 2023-03-01

**Authors:** Pengcheng Wang, Hai Yu, Henglin Xiao, Juan Wan, Qiang Ma, Gaoliang Tao, Qin Wang, Weiwei Jiang, Li Ma

**Affiliations:** 1 School of Civil Engineering, Architecture and Environment, Hubei University of Technology, Wuhan, China; 2 Innovation Demonstration Base of Ecological Environment Geotechnical and Ecological Restoration of Rivers and Lakes, Wuhan, China

**Keywords:** Mountainous slope, Ecological restoration, Species composition, Richness index, Slope position

## Abstract

This study investigated naturally-restored plant communities on wind farm slopes and analyzed the effects of various habitat factors on the plant diversity. The findings provide a technical support for the ecological restoration of mountainous slopes.Twenty-one slopes on five wind farms were selected and the characteristics of the habitat, including slope position, slope aspect, slope gradient, altitude, years since restoration, and plant communities, were recorded. The species richness of the plant communities and the vegetation diversity indexes of these wind farms were measured and calculated, including the Shannon-Wiener diversity index (*H*′), Pielou’s species evenness index (*J*), and Margalef’s richness index (*R*). The key factors influencing plant diversity were identified using a stepwise regression analysis. A total of 36 families, 54 genera, and 57 species of plants were identified in this study with the *Gramineae*, *Compositae*, *Rosaceae*, *Liliaceae*, and *Juglandaceae* families the mostly predominant. *Cynodon dactylon*, *Rubus lambertianus* Ser., and *Lindera glauca* were the dominant species of herbs, shrubs, and trees, respectively. The highest number of species were found on lower slopes, slopes with semi-sunny aspects, slopes with gradients 30–50°, elevation below 500 m, and on slopes with at least five years since restoration. The plant diversity *H*′ and *R* tended to be higher on lower slopes than on upper slopes, and higher on slopes with semi-shady aspects than on slopes with semi-sunny aspects (*P* < 0.05). Vegetation diversity increased with the years since restoration. Slope position and slope aspect were identified as the primary influencing factors, and the *H*′ and *R* indexes were major indicators of changes in plant diversity on mountainous slopes.

## Introduction

The rapid growth in economic development has meant an increase in large scale construction projects built on mountainsides. These developments in mountainous areas, including mining projects, road constructions, and wind farms, can lead to the disruption of the initial ecosystems and the destruction of the soil-plant systems (Squeo et al., 2016). Wind power is an important clean energy source. In China, wind power has surpassed nuclear power in energy capacity, becoming the third largest energy source, benefitting from broad market prospects (Zeng et al., 2013). As wind farm technology improves, mountainous regions with complex topography have become the primary locations for newly constructed, regional wind farms. However, these wind farm projects also include road construction and booster stations, damaging native vegetation and existing soil structure (Wang, 2009; Julian et al., 2017). The restoration and reconstruction of the ecosystem is conducive to improving and governing the extremely degraded ecological environment. Repairing damaged slopes is imperative to preventing geological disasters such as slope failure, landslides, and soil erosion. These disasters have seriously threatened to agricultural production and ecological safety (Dhar et al., 2020). Therefore, it is necessary to account for water-and-soil and ecological conservation and ensure that the existing plant diversity and ecosystem remain intact during the development and construction of wind farms (Guerra et al., 2017; Faiz, Ng & Rahman, 2022). Controlling soil erosion by improving soil fertilizer and structure is crucial for the restoration and reconstruction of slope ecosystems. Vegetation restoration requires a combination of both engineering and biological measures and begins with the restoration of plant diversity.

Many habitat factors impact the growth and diversity of vegetation (Girmay et al., 2020). During natural or artificial ecological restoration, regional climate conditions, topography and other habitat conditions have varying levels of impact on the diversity of plant communities (Siefert et al., 2012; Guo et al., 2019). A global meta-analysis showed that soil substrate was an important factor of roadside slope restoration actions (Wang, Liu & Pang, 2021). In the dryland area of Ethiopia, topographic variation associated with slope aspect and soil fertility should be considered for conservation measures (Girmay et al., 2020). Identifying the plant communities and its drivers could contribute to ecological restoration projects in a specific region (Squeo et al., 2016). In the disturbed areas surrounding wind towers in Romania, enforcing conservation activities had a more significant impact than controlling habitat factors on the recovery of plant communities and diversity (Urziceanu et al., 2021). There are many studies from China on the ecological restoration of side slopes at mines and river courses that have shown that a restoration strategy based on native herbs, shrubs and trees can successfully control soil erosion and create a stable slope (Keehn & Feldman, 2018). The slope gradient, intactness index of rock mass, slope aspect, and density of the structural plane are primary factors in the ecological restoration of mines (Hu et al., 2018). The introduction of species to the side slopes of open-pit mines should consider the habitat and climate conditions of the site (Chen et al., 2022). Variation in water level had a great impact on the restoration of vegetation in areas disturbed by river course projects in mountainous regions (Sheng et al., 2022). The diversity level of the existing plant community is an important indicator of the stability and resilience ability of the ecosystem. The types of species, quantity of species, and combination of species in the plant community can directly affect its structure, succession behavior, and diversity, affecting its ecological function (Urziceanu et al., 2021). Research on the effects of habitat factors on plant diversity during natural ecological restoration is important for the selection and configuration of plant species in side slope restoration projects. This study investigated naturally-restored side slopes of typical wind farms in Hubei province and analyzed the correlations between plant diversity and various habitat factors during ecological restoration at side slopes in mountainous regions. This study summarized the primary influencing factors of plant diversity on these slopes in mountainous regions, and its findings will help improving the efficiency of ecological restoration.

## Materials & Methods

### Overview of the study area

**Figure 1:**
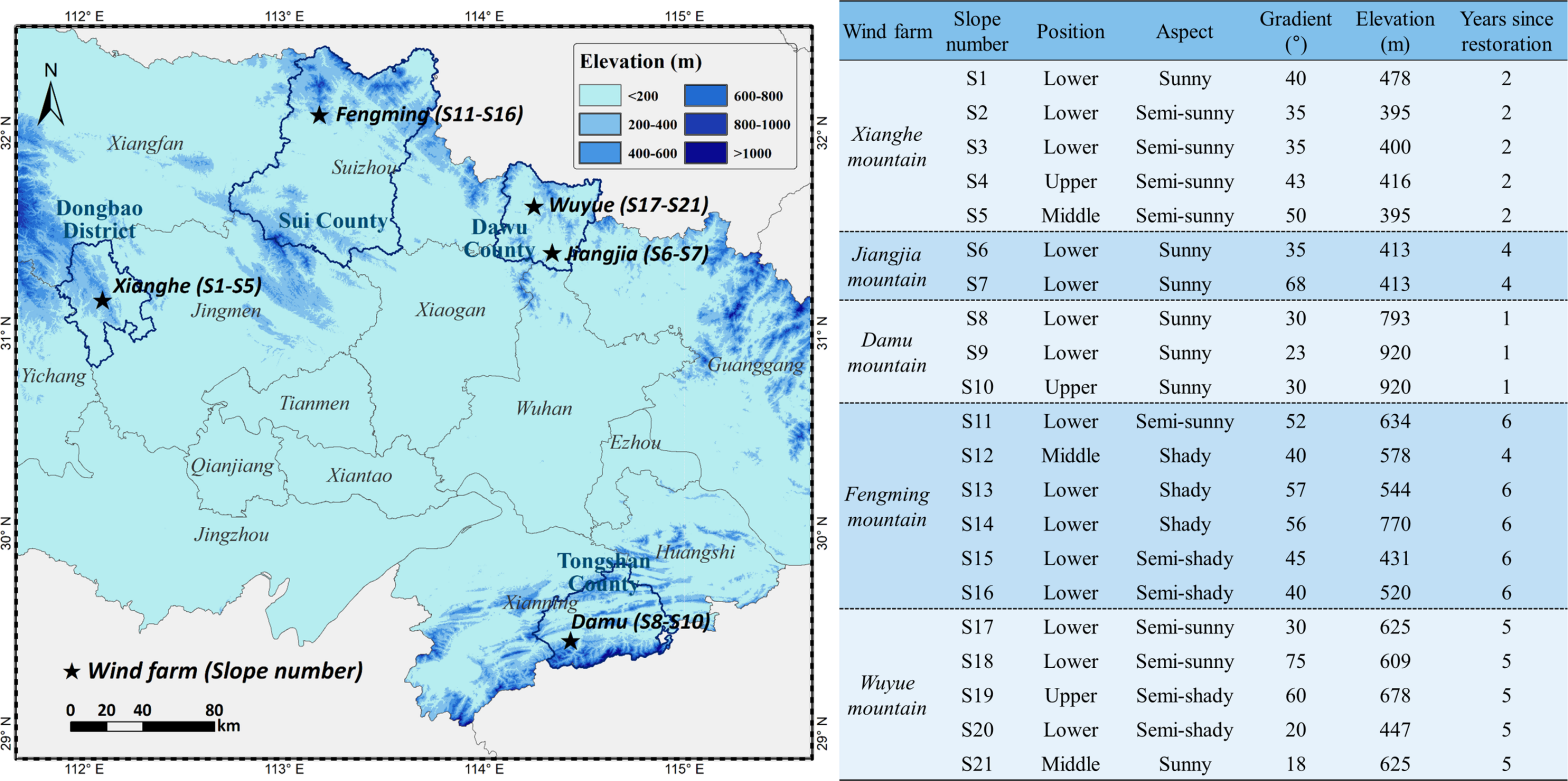
Relative frequency and abundance of plant species in the naturally-restored slopes.

The study area was located in four counties (or districts) of Hubei province China, namely Dongbao District, Dawu County, Tongshan County, and Sui County (Fig. 1). This area has a humid sub-tropical monsoon climate. The annual mean temperature, total sunshine hours, and total rainfall were 15.1 °C, 1950 h and 977 mm, respectively in Dongbao District; 15.3 °C, 2153 h and 1122 mm, respectively in Dawu County; 16.3 °C, 1845 h and 1500 mm, respectively in Tongshan County; and 15.4 °C, 2035 h and 968 mm, respectively in Sui County. The study area consists mostly of mountains and hills with a peak elevation of 684, 858, 954 and 1140 m in these four counties (or districts), respectively. The vegetation in the area is primarily evergreen broad-leaf forest and evergreen deciduous and broad-leaved mixed forest.

### Selection of sample plots

Five mountainous wind farms were selected from four counties (or districts) of the Hubei province: Xianghe, Jiangjia, Damu, Fengming and Wuyue (Fig. 1). In order to obtain representative data, 21 naturally-restored slope plots were investigated. These plots were comprised mainly of rock excavation and soil filling. The habitat factors of all 21 slopes are illustrated in Fig. 1.

### Selection of sample areas and investigation methods

This study focused on investigating the characteristics of side slope sample plots, including slope position, slope aspect, slope gradient, altitude, years since restoration and plant communities. Slope position can be divided into upper side slope (U), middle side slope (M) and lower side slope (L) from top to bottom, with each slope section’s height between 3 and 5 m. Slope aspect (with due north as 0°, degrees recorded clockwise) was divided into shady (337.6–67.5°), semi-shady (292.6–337.5°, 67.6–112.5°), semi-sunny (247.6–292.5°, 112.6–157.5°), and sunny (157.6–247.5°) slopes. The slope gradient and slope aspect were determined using hand-bearing a geological compass (ZN02; Jiesheng, Shenzen, China). The longitude, latitude, and altitude of the plots were determined using 3D GPS navigation (Xingyao X; Qianxun, Shanghai, China). The years since restoration was calculated starting from the completion of the restoration project in each area.

The sampling method for plant communities was as follows: in each sample plot, a 10 m × 10 m quadrat was set, and all the plants of tree species in the quadrant were investigated one by one. Each sample plot of tree species was then divided into four 5 m × 5 m quadrats, one of which was selected to investigate shrub species. Next, in each of the 5 m × 5 m quadrats, a 1 m × 1 m smaller quadrat was randomly chosen to investigate herbs species. The name, number of species and number of plants were recorded for each quadrat, and then the total number for the quadrant was calculate.

### Data processing and analysis

Relative frequency reflects the distribution of a plant species in a certain region. It is the ratio of the number of a species in a quadrant to the total number in the quadrant. Relative abundance reflects the amount of a plant species in the overall plant communities. It is measured as the ratio of the number of a certain plant species to the number of plants of all species.

In this study, three indexes were used for assessing the plant community, the Shannon-Wiener diversity index (*H*′), Pielou’s species evenness index (*J*) and Margalef’s richness index (*R*), which were calculated, respectively, using the formulas as below:

\begin{eqnarray*}& {H}^{{^{\prime}}}=-\sum {P}_{i}ln{P}_{i}(i=1,2,\ldots ,S) \end{eqnarray*}

\begin{eqnarray*}& J={H}^{{^{\prime}}}/lnS \end{eqnarray*}

\begin{eqnarray*}& R=(S-1)/\ln \nolimits N \end{eqnarray*}

where *S* represents the total number of plant species, *N* represents the total number of individual plants for all species, *N*_*i*_represents the numbers of each plant species, and *P*_*i*_ = *N*_*i*_/*N*.

The data were analyzed using Microsoft Excel (version 2013) and a map was created by ArcGIS Desktop (Version 10.0). The figures were drawn by Origin (version 2021) and the stepwise regression analysis was performed using SPSS (version 20.0).

## Results

### Species composition of plant communities on mountainous side slopes

The composition of the plant communities are as shown in Table 1. In the 21 sample plots, a total of 36 families, 54 genera,and 57 species were observed. The 14 families, 29 genera, and 30 species of herbs; 10 families, 12 genera, and 14 species of shrubs; and 12 families, 13 genera, and 13 species of trees identified. Of the 36 total families identified, *Gramineae* had 10 genera and 10 species, *Compositae* had five genera and six species, *Rosaceae* had four genera and six species, *Liliaceae* had two genera and two species, *Pteridiaceae* had two genera and two species, *Juglandaceae* had two genera and two species, and all other families had one genus and one species each. *Gramineae* was the largest family and accounted for 17.54% of the total species identified, followed by *Compositae* and *Rosaceae*, which each accounted for 10.53% of the total species.

**Table 1:** Relative frequency and abundance of plant species in the naturally restored slopes.

Life-form	Species	Family	Genus	Relative Frequency (%)	Relative Abundance (%)
Herb	*Cynodon dactylon*	*Gramineae*	*Cynodon*	42.86	38.82
	*Erigeron canadensis*	*Compositae*	*Erigeron*	42.86	8.81
	*Imperata cylindrica*	*Gramineae*	*Imperata*	38.1	12.74
	*Setaria viridis*	*Gramineae*	*Setaria*	33.33	34.16
	*Elsholtzia ciliata*	*Labiatae*	*Elsholtzia*	28.57	25.84
	*Miscanthus sinensis*	*Gramineae*	*Miscanthus*	23.81	3.16
	*Ophiopogon bodinieri*	*Liliaceae*	*Ophiopogon*	19.05	13.19
	*Allium macrostemon*	*Liliaceae*	*Allium*	14.29	25.61
	*Arthraxon hispidus*	*Gramineae*	*Arthraxon*	14.29	22.40
	*Rumex acetosa*	*Polygonaceae*	*Rumex*	14.29	17.68
	*Artemisia argyi*	*Compositae*	*Artemisia*	14.29	13.28
	*Artemisia apiacec*	*Compositae*	*Artemisia*	14.29	8.36
	*Phytolacca acinosa*	*Phytolaccaceae*	*Phytolacca*	14.29	4.9
	*Bolbitis cadieri*	*Pteridiaceae*	Bolbitis	14.29	4.53
	*Semiaquilegia adoxoides*	*Ranunculaceae*	*Semiaquilegia*	9.52	28.88
	*Eleusine indica*	*Gramineae*	*Eleusine*	9.52	21.26
	*Bidens bipinnata*	*Compositae*	*Bidens*	9.52	19.55
	*Hypolepis punctata*	*Pteridiaceae*	*Hypolepis*	9.52	3.46
	*Veronica didyma*	Scrophulariaceae	Veronica	4.76	32.61
	*Juncus effusus*	*Juncaceae*	*Juncus*	4.76	22.45
	*Chrysanthemum indicum*	*Compositae*	*Chrysanthemum*	4.76	17.80
	*Sporobolus fertilis*	*Gramineae*	*Sporobolus*	4.76	14.29
	*Lolium perenne*	*Gramineae*	*Lolium*	4.76	14.02
	*Gelsemium elegans*	*Loganiaceae*	*Gelsemium*	4.76	10.27
	*Daucus carota*	Umbelliferae	Daucus	4.76	9.35
	*Themeda triandra*	*Gramineae*	*Themeda*	4.76	7.79
	*Chloris virgata*	*Gramineae*	*Chloris*	4.76	3.57
	*Dicranopteris dichotoma*	*Gleicheniaceae*	*Dicranopteris*	4.76	0.93
	*Senecio scandens*	*Compositae*	*Senecio*	4.76	0.89
	*Solanum rostratum*	*Solanaceae*	*Solanum*	4.76	0.54
Shrub	*Pyracantha fortuneana*	*Rosaceae*	*Pyracantha*	14.29	17.24
	*Lonicera japonica*	*Caprifoliaceae*	*Lonicera*	14.29	21.36
	*Rubus lambertianus*	*Rosaceae*	*Rubus*	14.29	63.09
	*Camellia japonica*	*Theaceae*	*Camellia*	9.52	4.00
	*Rosa multiflora*	*Rosaceae*	*Rosa*	9.52	1.36
	*Smilax glabra*	*Liliaceae*	*Smilax*	4.76	7.14
	*Ligustrum lucidum*	*Oleaceae*	*Ligustrum*	4.76	11.90
	*Rubus hirsutus*	*Rosaceae*	*Rubus*	4.76	7.78
	*Acer cordatum*	*Aceraceae*	*Acer*	4.76	7.14
	*Rubus coreanus*	*Rosaceae*	*Rubus*	4.76	5.36
	*Lespedeza bicolor*	*Leguminosae*	*Lespedeza*	4.76	1.79
	*Rhododendron simsii*	*Ericaceae*	*Rhododendron*	4.76	1.39
	*Nandina domestica*	*Berberidaceae*	*Nandina*	4.76	1.39
	*Nerium indicum*	*Apocynaceae*	*Nerium*	4.76	0.93
Tree	*Lindera glauca*	*Lauraceae*	*Lindera*	80.95	3.01
	*Pinus masoniana*	*Pinaceae*	*Pinus*	33.33	1.72
	*Platycarya strobilacea*	*Juglandaceae*	*Platycarya*	14.29	2.31
	*Photinia scrrulata*	*Rosaceae*	*Photinia*	9.52	5.41
	*Sabina chinensis*	*Cupressaceae*	*Juniperus*	4.76	11.36
	*Cunninghamia laceolata*	*Taxodiaceae*	*Cunninghamia*	4.76	9.09
	*Rhus Chinensis*	*Anacardiaceae*	*Rhus*	4.76	6.82
	*Ulmus pumila*	*Ulmaceae*	*Ulmus*	4.76	4.11
	*Zanthoxylum bungeanum*	*Rutaceae*	*Zanthoxylum*	4.76	2.78
	*Acer mairei*	*Juglandaceae*	*Pterocarya*	4.76	2.27
	*Castanea mollissima*	*Fagaceae*	*Castanea*	4.76	1.11
	*Ilex chinensis*	*Aquifoliaceae*	*Ilex*	4.76	1.05
	*Aleurites fordii*	*Euphorbiaceae*	*Vernicia*	4.76	0.93

The calculations of the relative frequency and the abundance of species found that, *Cynodon dactylon* had the highest relative frequency (42.86%) and relative abundance (38.82%),followed by *Setaria viridis*, *Allium macrostemon*, *Ophiopogon bodinieri*, *Erigeron canadensis*, *Artemisia argyi*, and *Rumex acetosa*. Among shrubs, *Rubus lambertianus* had the highest frequency (14.29%) and relative abundance (63.09%), followed by *Lonicera japonica* and *Pyracantha fortuneana*. Among trees, *Lindera glauca* was predominant in terms of relative frequency (80.95%), followed by *Pinus massoniana* (33.33%) and *Platycarya strobilacea* (14.29%).

### Species richness under different habitat conditions

**Figure 2:**
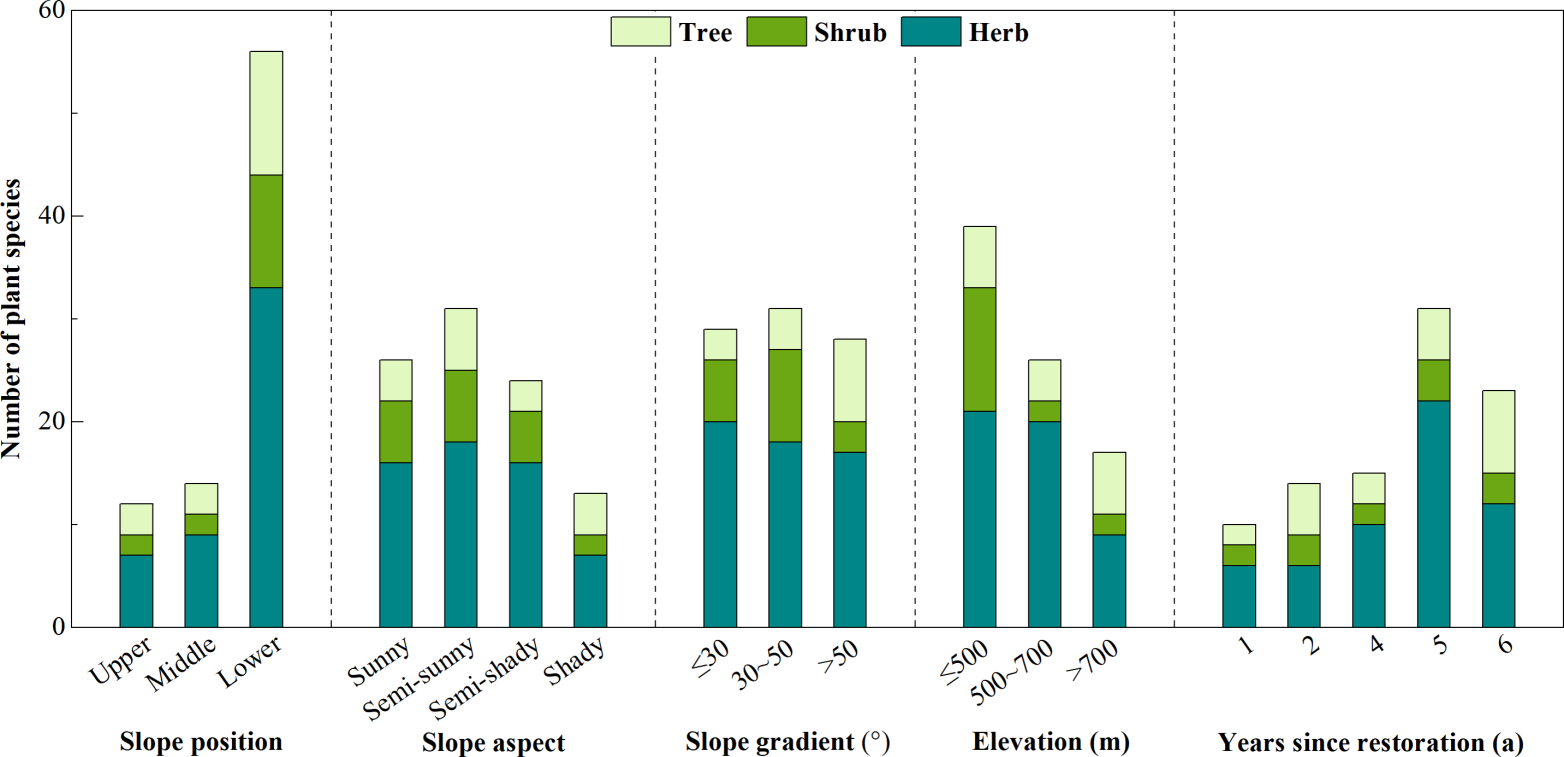
The number of plant species on side slopes under different habitat conditions.

As shown in Fig. 2, under different slope positions, the total number of plant species decreased from the upper slope to the lower side slopes, with the lower side slopes having 4.00 times as many plants species as the middle side slopes and 4.67 times as many species as the upper side slopes. The species number of herbs, shrubs, and trees were all the most on lower side slopes with 33, 11, and 12 species identified on these slopes, and were all the least on upper side slopes with seven, two and three species identified, respectively. The total number of plant species also followed a descending order among semi-sunny, sunny, semi-shady, and shady side slopes. The total number of plant species identified on semi-sunny side slopes was 19.23%, 24.00%, and 93.75% higher than on sunny, semi-shady, and shady side slopes, respectively. The total number of herbs, shrubs, and tree species identified were all the most numerous on semi-sunny side slopes with the 18, seven, and six species identified, respectively. Species of herbs and shrubs were the least numerous on shady side slopes with seven and two species identified, respectively. The total number of plant species was the highest at the slope gradient between 30° and 50°, with this slope gradient having 16.7% more species than other slope gradients. Herbs were the most numerous on side slopes with slope gradients less than or equal to 30° with at 30 species of herbs identified on these slopes. Species of herbs and shrubs were the least numerous on side slopes with slope gradients less than 50° with 17 species of herbs and three species of shrubs identified on these slopes. Altitude also impacted the total number of plant species, with the largest number of species identified on slopes in this altitude range was 50.00% higher than on slopes between 500 and 700 m and 116.67% higher than on side slopes above 700 m in altitude. Species of herbs, shrubs, and trees were all the most numerous on side slopes less than or equal to 500 m in altitude with 21 species of herbs, 12 species of shrubs, and 6 tree species identified on these slopes. Species of herbs and shrubs were the least numerous on side slopes above 700 m in altitude with nine herb species and two shrub species identified at this altitude. The recovery period also impacted plant diversity with the total number of plant species increasing with the years since restoration. Species of herbs, shrubs, and trees were all the least numerous when there had been less than one year since restoration, with six herb species, two shrub species, and two tree species identified. Species of herbs and shrubs were the most numerous when there had been 5 or 6 years since restoration.

**Figure 3:**
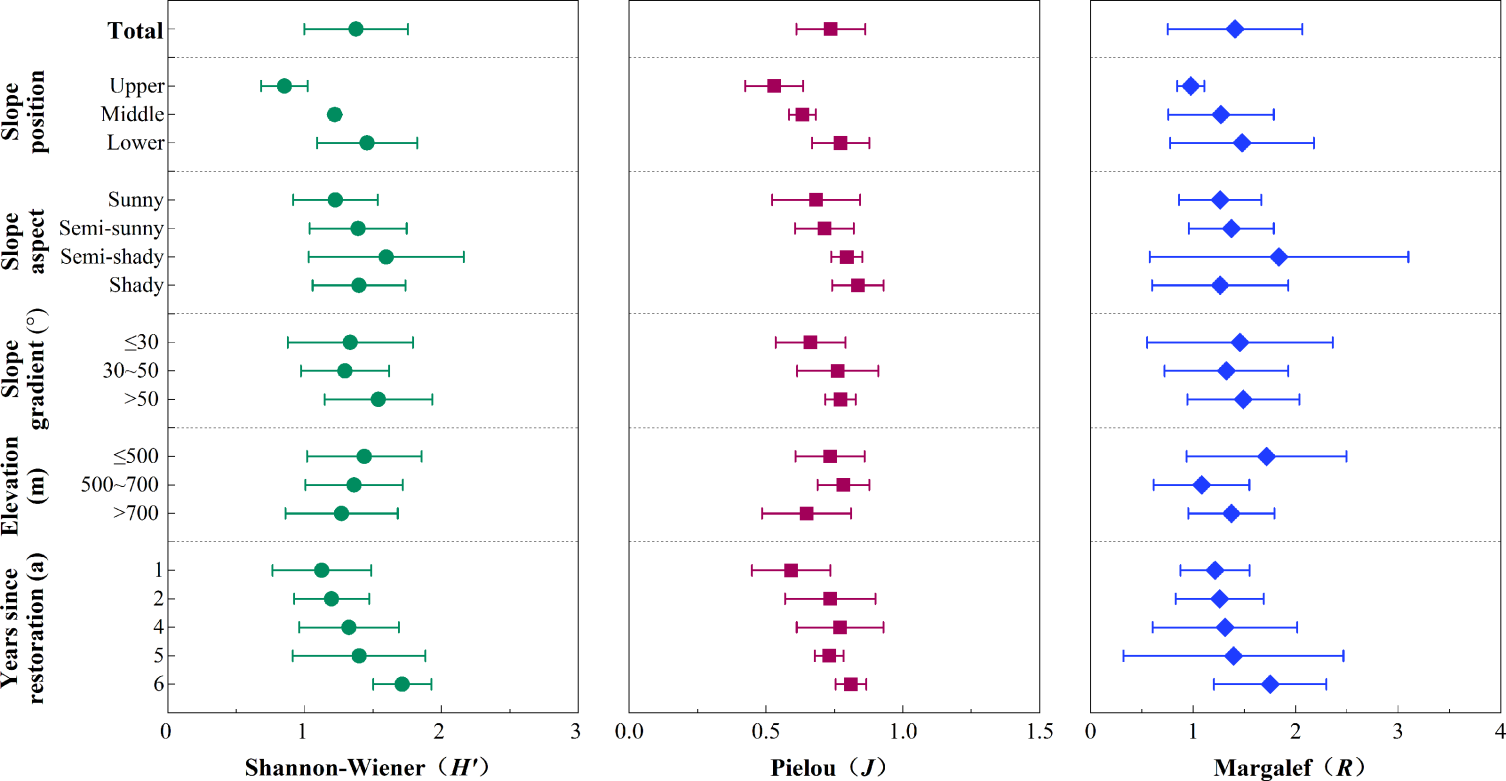
Effect of habitat factors on vegetation diversity index.

### Vegetation diversity under different habitat conditions

As shown in Fig. 3, Shannon-Wiener diversity index (*H*′), Pielou’s species evenness index (*J*), and Margalef’s richness index (*R*) decreased with side slope position (*P* < 0.05), with the variation ranges at 0.84−1.51 on lower slide slopes, 0.60−0.75 on middle side slopes, and 0.82−1.64 on upper slide slopes. All the indexes peaked on lower side slopes with values 79.84%, 24.73%, and 100.36% higher than upper side slopes, respectively, and 30.95%, 2.32%, and 59.22% higher than middle side slopes, respectively. The Shannon-Wiener diversity index (*H*′) and Margalef’s richness index (*R*) were more influenced by slope aspect, with both indexes *H*′ and *R* reaching their minimums on sunny side slopes (1.20 and 1.26) and their maximums on semi-shady side slopes (1.60 and 1.84). Both indexes were significantly higher on the semi-shady slopes than the sunny side slopes (*P* < 0.05). Pielou’s species evenness index (*J*) reached its maximum (0.84) on shady side slopes, which was 25.37% higher than on sunny side slopes. Generally, *H*′ and *R* steadily increased with increased years since restoration. *H*′, *J,* and *R* all reached their maximums after six years had passed since restoration, with index level 22.39%, 17.81%, and 25.17% higher than after only one year since restoration, respectively. In this study, there were no obvious variation trends in the plant diversity indexes with changes in the side slope gradient and altitude.

The stepwise regression analysis model and its parameters are shown in Table 2. The stepwise regression of the five habitat factors found that the main influencing factors of Shannon-Wiener diversity index (*H*′) and Pielou’s species evenness index (*J*) were side slope position and aspect, while the main influencing factor on Margalef’s richness index (*R*) was side slope aspect. The regression coefficients of the years since restoration, slope gradient, and altitude were so small that they were excluded. Therefore, side slope positions and aspects were the key factors influencing plant diversity, while other factors had no significant effect on plant diversity.

**Table 2:** Plant diversity measurement indexes and the regression model under different habitat factors. *Y* represents the diversity index, *X*_1_ represents the side slope position, and *X*_2_ represent side slope aspect.

Diversity indexes	Regression model	R^2^	F	*P*
*H*′	*Y* = 0.204+ 0.343*X*_1_+ 0.128*X*_2_	0.564	11.66	<0.001
*J*	*Y* = 0.441+ 0.051*X*_1_+ 0.068*X*_2_	0.315	4.138	<0.05
*R*	*Y* = 0.299+ 0.442*X*_1_	0.247	6.217	<0.05

## Discussion

This study shows that during the natural ecological restoration of side slopes in the study area, plants of families *Gramineae*, *Compositae*, *Rosaceae*, *Liliaceae*, and *Juglandaceae* were the most predominant, which is consistent with previous findings (Sheng et al., 2015). *Cynodon dactylon*, *Rubus lambertianus,* and *Lindera glauca* were the predominant species of herbs, shrubs, and trees, respectively. *Cynodon dactylon* has strong stress resistance, including being drought-resistant, cold-resistant, and poor-soil-fertility-resistant, and its seeds are easy to disperse, which makes it a pioneer plant in ecological restoration projects (Singh, Pandey & Singh, 2013). As a common warm-season species in natural environment, it is always utilized with *Lolium perenne*, *Poa annua,* and other cold-season plants (Hou et al., 2020). *Rubus lambertianus* is a semi-deciduous vining shrub, and rarely used in side slope restoration projects (Zhang et al., 2021). *Lindera glauca* is a deciduous shrub whose leaves persist into winter and do not fall until young leaves grow in the next year. It is usually used to construct deciduous forest landscape dominated by autumn and winter scenery (Chen et al., 2016). In this study, *Pinus massoniana* was the predominant tree species. As an evergreen tree, *Pinus massoniana* has strong adaptability and is often used in restoration projects in areas with a constant landscape in all four seasons (Zhao et al., 2021). Vegetation restoration projects using *Lindera glauca* and *Pinus massoniana* as dominat plants are more effective in improving soil water retention, organic matter content, and microbial community structure (Dou et al., 2013; Yu et al., 2022). Identifying the dominant plant species in natural ecological restoration projects provides significant reference value for the selection and configuration of plant species in side slope restoration.

The Shannon-Wiener diversity index (*H*′), Pielou’s species evenness index (*J*), and Margalef’s richness index (*R*) can quantitatively reflect the species heterogeneity in a competitive environment (Zhu et al., 2007). In this study, *H*′ and *R* were major indicators of changes in the of plant communities, and were significantly affected by side slope characteristics. The *J* had a lower indication value and was less influenced by habitat factors (Zou et al., 2013). Affected by mountain terrains of the study area, the temperature and humidity of local habitat varied greatly, and the processes and intensities of soil development changed accordingly changing the structure of plant communities (Lenoir et al., 2013). These changes may be an effect of the different gradients of mountainous slopes as well as side slope position. Plant diversity on lower slopes was significantly higher than middle and upper slope, and was possibly correlated with soil moisture, nutrient conditions, and illumination intensity (Shovon et al., 2020). A previous study suggested that there was a significant positive correlation between the *H*′ and *R* of alpine grassland species and the organic matter, total nitrogen, total phosphorus in soil, and lower side slopes were superior to both upper side slopes and middle side slopes in terms of soil nutrient, soil moisture, and temperature (Oztas, Koc & Comakli, 2003). Therefore, lower side slopes were more conducive to the growth of plants and had higher plant diversity (Méndez-Toribio et al., 2017). The slope aspect of side slopes also influencing habitat factors including illumination, wind speed, wind direction, soil moisture, and soil nutrient, which resulted in differences in plant diversity (Xue et al., 2018). For example, compared with shady side slopes, sunny side slopes had higher temperature, a greater evaporation capacity, lower humidity, greater soil weathering, lower organic matter contents, and were more likely to have drought and infertile condition (Qin et al., 2019). In this study, *H*′ and *R* indexes for different side slope aspects decreased among semi-shady, shady, semi-sunny, and sunny side slopes, while a previous study on an alpine meadow plant community in East Qilian Mountains found decreases in the *H*′ and *R* indexes among shady, sunny, semi-sunny, and semi-shady side slopes (Zhang et al., 2019). Improving plant communities through ecological restoration should account for both the site conditions of the side slope as well as regional differences in habitat conditions (Young, Petersen & Clary, 2010).

As the time since restoration increases, the soil erosion coefficient decreases significantly while moisture, organic matter, and total nitrogen content increases, which all help improve the growth of plants and increase plant diversity (Arunachalam & Pandey, 2003; Zhu et al., 2010). This study also revealed that the three different plant diversity measurement indexes, *H*′, *J,* and *R* all increased with the time since restoration (Wang et al., 2019), but were not impacted by changes in side slope gradient or elevation. In gentle side slope areas, soil layers are generally deeper with higher water and nutrient content, while soil layers in steep side slope areas were usually have better drainage (Liu et al., 2022). The elevation of side slopes affects the geographical structure and hydrothermal process of mountainous ecological system (Wang et al., 2007). As a result, plant diversity in this study decreased as elevation increased, including that the plants in this study had greater adaptability to different side slope gradient and elevation conditions (Wu et al., 2021).

This study investigated the plant species and habitat factors of 21 slopes. However, some uncontrollable factors, like the selection of slope and habitat factor, may add limitations to the results of this study. The selection of the naturally-restored slopes used in this study was sometimes subjective, but the result was consistent with expectation and experience. In future studies, the descriptions of the chosen slopes could be more detailed, and the distribution of sample plots at different sites could be more reasonable and uniform. In this study, we did not choose the same number of sample plots on each slope since the distribution of habitat factors were generally representative. The results of this study provide a technical support for ecological restoration projects in mountainous slopes. We believe that these results are consistent with those of similar climatological regions, and the analytical method used in this study is applicable to other slopes.

## Conclusions

During the natural restoration of side slopes in mountainous wind farms, plant families like *Gramineae*, *Compositae*, *Rosaceae*, *Liliaceae*, and *Juglandaceae* showed better adaptability to the environment. The predominant species of herbs, shrubs, and trees identified in this study were *Cynodon dactylon*, *Rubus lambertianus,* and *Lindera glauca*, respectively. The Shannon-Wiener diversity index (*H*′), Pielou’s species evenness index (*J*), and Margalef’s richness index (*R*) were primary indicators of variation in plant communitiesin the side slopes studied. There was wide range of variation based on the characteristics of the side slope. Among the five habitat factors studied, slope position and aspect were the primary factors influencing plant diversity. The number of years since restoration had some positive effects on plant community, whereas, slope gradient and elevationdid not significant affect plant diversity. Prior to the ecological restoration of side slopes wind farms, it is important to select plant species based on the slope conditions and consider the local dominant species for the best restoration result.

##  Supplemental Information

10.7717/peerj.14912/supp-1 Data S1 Raw data

## Additional Information and Declarations

### Data Availability

The following information was supplied regarding data availability: The raw measurements are available in the Supplementary File.
